# Le syndrome des loges du bras: une complication inhabituelle de l'intoxication au monoxyde de carbone

**DOI:** 10.11604/pamj.2015.20.405.6675

**Published:** 2015-04-24

**Authors:** Khalid Chkoura, Hicham Kechna, Jaouad Loutid, Omar Ouzad, Toufiq Cherradi, Moulay Ahmed Hachimi

**Affiliations:** 1 Pôle d'Anesthésie Reanimation et Urgences, Hôpital militaire d'instruction Moulay Ismail, Meknès, Maroc; 2 Service de Traumatologie et Orthopédie, Hôpital militaire d'instruction Moulay Ismail, Meknès, Maroc

**Keywords:** Syndrome des loges du bras, monoxyde de carbone, intoxication, Compartment syndrome of the arms, carbon monoxide, intoxication

## Abstract

Le monoxyde de carbone (CO) surnommé “Silent killer” par les Anglo-Saxons représente la première cause de mortalité par intoxication accidentelle ou volontaire en Europe comme aux États-Unis. Au Maroc, le centre anti poison a collecté entre 1991 et 2008, 12976 cas d'intoxication au monoxyde de carbone dont 98,7% étaient accidentelles. Cette intoxication est très exceptionnellement compliquée d'un syndrome de loge qui peut contribuer à une aggravation certaine du pronostic fonctionnel et vital quand il est ignoré ou dominé par d'autres symptômes en particulier neurologiques. Nous rapportons le cas d'un gardien de nuit qui a présenté un syndrome de loge particulier par sa localisation (bras) au cours d'une intoxication au CO qui a évolué favorablement.

## Introduction

Le monoxyde de carbone (CO) est un gaz inodore, incolore, non irritant et sans saveur lui donnant un caractère insidieux «Silent killer». L'intoxication au CO demeure la première cause de morbidité et de mortalité d'origine toxique dans le monde. Elle représente un véritable problème de santé publique [1, 2]. Le syndrome de loge est une complication exceptionnelle mais qui peut être grave de cette intoxication.

## Patient et observation

**Figure 1:**
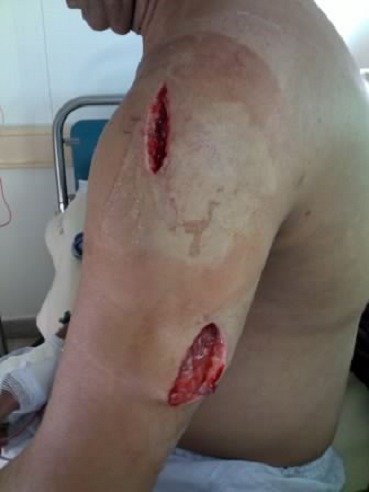
Aponévrotomies au niveau du bras et de la région deltoïde

Mr. HS âgé de 53 ans, tabagique chronique à 32 paquets année, pesant 83 kg pour une taille de 171cm gardien d'un magasin est admis au service d'accueil des urgences pour une intoxication au monoxyde de carbone avec traumatisme crânien. Il est retrouvé le matin d'une journée de l'hiver allongé en décubitus latéral, l’épaule gauche comme appui, inconscient avec une petite plaie du cuir chevelu en présence d'un braséro avec de la cendre. A son admission, il était somnolent et accusait des céphalées. Il était apyrétique, sa tension artérielle à 123/71 mmHg, sa fréquence cardiaque à 89 battements par minute. Par contre il était polypneïque à 19 cycles par minute et sa saturation à l'air ambiant était à 88%. L'examen clinique objectivait une tuméfaction de l’épaule gauche qui s’étendait au thorax et au bras. L'examen de la région deltoïde et brachiale trouvait une douleur importante avec une tension de la loge deltoïde et des loges antérieure et postérieure du bras. Les pouls radial et cubital étaient présents et symétriques. L'examen neurologique a mis en évidence un syndrome cérébelleux plus une hypoesthésie du moignon de l’épaule gauche avec une anesthésie des faces dorsale et palmaire de la main. Les radiographies de l’épaule, du poumon ainsi que le scanner cérébral étaient sans anomalie. L’électrocardiogramme était normal. Par ailleurs, la recherche de toxiques dans le sang, les urines et le liquide gastrique était négative. Le bilan biologique notait une hyperleucocytose à 16900 éléments par mm3, une CRP à 90 mg/l, un taux des créatines phosphokinases à 34074 UI/l en plus d'une insuffisance rénale avec un taux d'urée à 0.75 g/l et une créatininémie à 31 mg/l. Au total il s'agissait d'une intoxication grave au CO compliquée d'un syndrome de loge de l’épaule et du bras du membre supérieur gauche avec une souffrance neurologique du plexus brachial. L’évolution était favorable sous oxygénothérapie à haut débit, plus une analgésie adaptée et une réhydratation forcée associées à deux aponévrotomies: une au niveau de la région deltoïde et l'autre au niveau brachial (Figure 1).

## Discussion

Le monoxyde de carbone (CO) surnommé Silent killer par les Anglo-Saxons représente la première cause de mortalité par intoxication accidentelle ou volontaire en Europe comme aux États-Unis [1]. Au Maroc, le centre anti poison a collecté entre 1991 et 2008, 12976 cas d'intoxication au CO dont 98,7% étaient accidentelles. Les manifestations musculosquelettiques étaient rares et n'ont représenté que 0,1% des cas. Par contre les signes neurologiques ont représenté 28%, [2]. L'apparition d'un syndrome de loge au cours d'une intoxication au CO est une situation très rare. Le premier ayant décrit cette association était Larrey en 1812 chez les soldats de Napoléon au cours de l'occupation de Berlin, puis elle a été décrite dans d'autres publications. Le syndrome de loge se manifeste typiquement par la douleur, la tension et les signes déficitaires en rapport avec les nerfs passant à travers cette loge qui sont très caractéristiques et qui sont les signes les plus fiables pour le diagnostic positif et topographique. Cependant, La présence de troubles de conscience secondaires à l'intoxication au CO peut rendre le diagnostic difficile et les signes orientant vers le syndrome de loge sont ceux de la rhabdomyolyse. Sa localisation au niveau du bras est très rare. Ceci est attribué à la souplesse de l´aponévrose du bras et à la rareté des grandes structures tendineuses et ligamentaires à ce niveau. En plus, les compartiments du bras communiquent avec ceux de la ceinture scapulaire donnant plus d´espace pour la distribution de l'oedème [3]. Orizaga M. a rapporté deux cas de syndrome de loge associés à une intoxication au CO dont un qui intéressait l'avant bras et le bras avait un caractère positionnel [4].

Par ailleurs, Abdul-Ghaffar N. a rapporté cinq cas d'empoisonnement au CO. Deux victimes sont décédées sur la scène et les trois autres survivants ont présenté un ou plusieurs syndromes de loges. La durée de leur exposition était trop longue dans une petite chambre confinée faisant penser que la rhabdomyolyse ainsi que le syndrome de loge qu'il soit postural ou non sont l’évolution inéluctable de toute intoxication au CO [5]. L'association de rhabdomyolyse avec l'intoxication au monoxyde de carbone est décrite par plusieurs auteurs. Cette rhabdomyolyse intéressait plusieurs groupes musculaires et semble être liée à une durée d'exposition au CO trop longue plus qu’à un caractère postural. En conséquence, outre le caractère postural prolongé qui peut expliquer la survenue d'un syndrome de loge au cours d'une intoxication au CO, un deuxième mécanisme peut être en cause, c'est la rhabdomyolyse en rapport avec l'hypoxie et la toxicité cellulaire engendrées par le CO [6]. Plusieurs mécanismes peuvent être responsables de la rhabdomyolyse au cours de l'intoxication au CO, l'hypoxie périphérique due à une diminution du transport d'oxygène par l'hémoglobine et la formation de l'HbCO, la toxicité directe du CO sur les cellules musculaire par fixation du CO sur la myoglobine (MbCO) ainsi que par la formation des espèces radicalaires de l'oxygène.

Cependant, la rhabdomyolyse au cours d'une intoxication au CO n'est pas toujours synonyme de l'installation de syndrome de loge. Par contre, un oedème sous-aponévrotique est rapporté presque dans tous les cas de rhabdomyolyse avec intoxication au CO et peut être impliqué dans l'apparition de syndrome de loge et de gangrènes. Le traitement du syndrome de loge secondaire au cours d'une intoxication oxycarbonée est une urgence médico-chirurgicale. Après avoir évacué le patient, il faut doser si possible la concentration de CO dans l'atmosphère, trouver la source pour l'arrêter, aérer le local et commencer l'oxygénothérapie au masque. L'oxygène peut être administré à pression normale au masque à haute concentration 12 à 15 litres/ minute ou à pression élevée dans des chambres hyperbares. L'aponévrotomie décompressive doit être réalisée dans les plus brefs délais pour limiter au mieux les séquelles parfois gravissimes, au plus tard dans les 6 premières heures par nécessité devant un syndrome de loge évident cliniquement et/ou sur le résultat des mesures de pression. “Mieux vaut opérer pour rien que de laisser évoluer un syndrome de loge” [3].

## Conclusion

Bien qu'il soit exceptionnellement décrit au cours des intoxications au monoxyde de carbone, la présence d'un syndrome de loge pourrait aggraver le pronostic quand il est tardivement pris en charge.
